# Inferred vs Realized Patterns of Gene Flow: An Analysis of Population Structure in the Andros Island Rock Iguana

**DOI:** 10.1371/journal.pone.0106963

**Published:** 2014-09-17

**Authors:** Giuliano Colosimo, Charles R. Knapp, Lisa E. Wallace, Mark E. Welch

**Affiliations:** 1 Biological Sciences, Mississippi State University, Mississippi State, Mississippi, United States of America; 2 Daniel P. Haerter Center for Conservation and Research, John G. Shedd Aquarium, Chicago, Illinois, United States of America; Institute of Biochemistry and Biology, Germany

## Abstract

Ecological data, the primary source of information on patterns and rates of migration, can be integrated with genetic data to more accurately describe the realized connectivity between geographically isolated demes. In this paper we implement this approach and discuss its implications for managing populations of the endangered Andros Island Rock Iguana, *Cyclura cychlura cychlura*. This iguana is endemic to Andros, a highly fragmented landmass of large islands and smaller cays. Field observations suggest that geographically isolated demes were panmictic due to high, inferred rates of gene flow. We expand on these observations using 16 polymorphic microsatellites to investigate the genetic structure and rates of gene flow from 188 Andros Iguanas collected across 23 island sites. Bayesian clustering of specimens assigned individuals to three distinct genotypic clusters. An analysis of molecular variance (AMOVA) indicates that allele frequency differences are responsible for a significant portion of the genetic variance across the three defined clusters (F*_st_* =  0.117, p
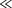
0.01). These clusters are associated with larger islands and satellite cays isolated by broad water channels with strong currents. These findings imply that broad water channels present greater obstacles to gene flow than was inferred from field observation alone. Additionally, rates of gene flow were indirectly estimated using BAYESASS 3.0. The proportion of individuals originating from within each identified cluster varied from 94.5 to 98.7%, providing further support for local isolation. Our assessment reveals a major disparity between inferred and realized gene flow. We discuss our results in a conservation perspective for species inhabiting highly fragmented landscapes.

## Introduction

Natural or anthropogenic habitat fragmentation may hinder or prevent animal dispersal. Inherently linked to dispersal, gene flow is also largely determined by geographic features such as fragmentation [1]. High rates of dispersal and gene flow, favor genetic homogenization across broad geographic ranges. In spite of high dispersal, a lack of gene flow between geographic isolates enhances the likelihood of local adaptation, random loss of genetic variability, and reduction in population size, which increase the probability of inbreeding depression and local extinction [2]–[6].

Though life history data and field observations provide important insights regarding patterns of species dispersal, it is imperative that potential differences in perceived and realized gene flow be recognized if we are to implement effective conservation measures. For instance, adopting translocation as a strategy to reinforce declining populations can be harmful if the source population is genetically distant from the recipient one due to historical lack of gene flow [7]. Moreover, inferring gene flow and dispersal based on landscape features alone can erroneously represent how genetic variation is spatially structured. For example, molecular and landscape analyses in different species of salamanders (genus *Ambystoma*) revealed unexpected high connectivity between geographically isolated demes [8]. These salamanders have the ability to migrate between ponds via habitats characterized by relatively high dispersal costs, hence reducing genetic differences between isolated ponds [1]. Species ecology also affects our perception of gene flow. It is expected, for example, that flight of highly mobile organisms like birds could mitigate differentiation between adjacent populations [9]. Bertrand and colleagues [10], however, demonstrated that even at short distances (<26 km), groups of island passerine birds, *Zosterops borbonicus*, exhibited extremely reduced rates of gene flow. Indeed, perceived migration rates fell short of realized gene flow rates determined by population genetic analyses. This discrepancy could be exacerbated in highly fragmented landscapes, and in species of conservation concern, could lead to misguided management. In this paper we use molecular tools to investigate the conformity between inferred and realized patterns of gene flow in the endangered Andros Island Rock Iguana, *Cyclura cychlura cychlura*.

**Figure 1:**
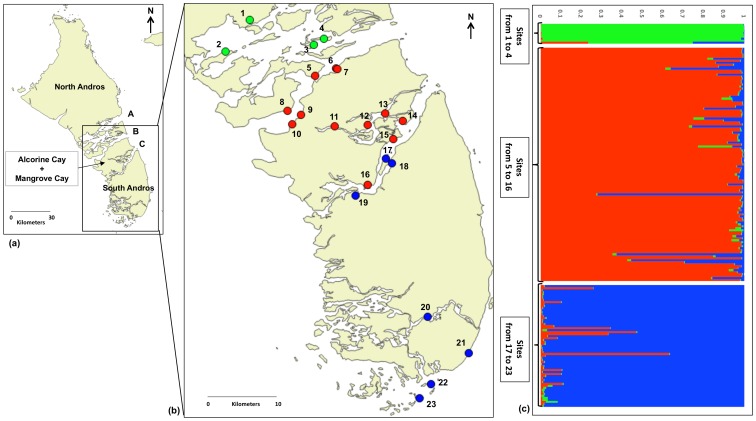
Map of Andros Island depicting its four major landmasses, the sampling locations and the assignment test results. (**a**) North Andros, Alcorine Cay, Mangrove Cay and South Andros. A, B and C indicate North, Middle and South bight respectively (the map is not resolved enough to depict the separation of Mangrove and Alcorine Cays by Lisbon creek); (**b**) A map of the 23 sampling locations (see Table 1 for details on each location). Sampling sites are colored according to the predominant assignment of individuals to one of three populations given the result of the STRUCTURE analysis; (**c**) Bayesian clustering output.

The Andros Rock Iguana is endemic to Andros Island in the Bahamas [11], which is a composite of four major landmasses and hundreds of satellite cays separated by water channels, called bights, and smaller saline creeks (Figure 1a). Further, the island is composed of multiple habitat types including pine woodlands with open or closed broadleaf understory, dry evergreen scrublands, and intertidal mangroves [12], [13]. Despite this naturally fragmented and mosaic landscape, dispersal and gene flow may be high in the Andros Iguana. There is anecdotal and documented evidence of this species dispersing across water channels [12]. Additionally, *C. c. cychlura* hatchlings have been documented to readily disperse from nesting areas across a variety of different habitat types [14]. This system provides a valuable opportunity to investigate the conformity between perceived and realized migration patterns and to estimate the relative importance of different types of barriers to gene flow.

Current conservation plans for the Andros Island Rock Iguana [15] were informed largely by a long-term ecological study of the species [13], [14], [16], [17]. In the present study we expand on these investigations to infer metapopulation dynamics of these iguanas using neutral molecular markers. We used 16 polymorphic microsatellites to define the population genetic structure of *C. c. cychlura* on Andros Island. We then examined the population structure in the context of the species ecology and the associated landscape to infer patterns of gene flow. We hypothesized that the high dispersal rates inferred from anecdotal accounts and telemetry studies [12], [14] limit genetic divergence among local demes of *C. c. cychlura* on Andros Island despite their patchy distribution across geographically isolated sites. If true, we anticipate limited evidence for genetic structure across the island.

## Results

One hundred ninety two individuals were sampled from 23 sites. Identifiers for each sampled location and numbers of individuals sampled per site are listed in Table 1. Due to the extremely low density of iguanas in the northern region, no captures were possible on North Andros. Sample sizes reflect exhaustive efforts to sample animals. Low sample numbers indicate extremely low density, not sampling effort.

**Table 1:** Sampling site details.

Site ID	Name	N	H*_o_*	H*_e_*	HWE p-val
1	North Middle Bight 1	4	0.525 (0.26)	0.435 (0.167)	ns
2	North Middle Bight 2	1	-	-	-
3	North Middle Bight 3	4	0.568 (0.315)	0.575 (0.122)	ns
4	North Middle Bight 4	1	-	-	-
5	North Middle Bight 5	2	0.700 (0.421)	0.700 (0.131)	ns
6	North Middle Bight 6	4	0.461 (0.431)	0.557 (0.132)	ns
7	Mangrove Alcorine 1	1	-	-	-
8	North Middle Bight 7	4	0.571(0.284)	0.625(0.171)	ns
9	Mangrove Alcorine 2	1	-	-	-
10	Mangrove Alcorine 3	37	0.575 (0.268)	0.551 (0.260)	ns
11	Mangrove Alcorine 4	5	0.723 (0.265)	0.664 (0.175)	ns
12	Mangrove Alcorine 5	36	0.523 (0.207)	0.569 (0.185)	***
13	Mangrove Alcorine 6	4	0.613 (0.264)	0.654 (0.136)	ns
14	Mangrove Alcorine 7	6	0.547 (0.224)	0.615 (0.136)	ns
15	Mangrove Alcorine 8	7	0.615 (0.266)	0.576 (0.205)	ns
16	Mangrove Alcorine 9	10	0.564 (0.231)	0.577 (0.180)	ns
17	South Andros 1	36	0.594 (0.230)	0.581 (0.183)	ns
18	South Andros 2	4	0.569 (0.105)	0.639 (0.110)	ns
19	South Andros 3	4	0.533 (0.296)	0.617 (0.210)	ns
20	South Andros 4	4	0.553 (0.262)	0.609 (0.178)	ns
21	South Andros 5	2	0.692 (0.253)	0.666 (0.180)	ns
22	South Andros 6	7	0.564 (0.273)	0.505 (0.192)	ns
23	South Andros 7	4	0.440 (0.260)	0.550 (0.191)	ns

Name, number of individuals (N), observed (H*_o_*(s.e.)) and expected heterozygosity (H*_e_*(s.e.), and significant departure from HWE for each sampling site (ns  =  non significant; ***  =  significance at p = 0.01).

Of 23 microsatellite markers developed in congeneric *Cyclura* species and analyzed, 18 were successfully amplified, and 13 were polymorphic in *C. c. cychlura* (Table S1). In addition, we successfully designed three species-specific polymorphic markers. Of 93 sequenced inserts, 15 contained short tandem repeats and were used for designing species-specific microsatellite primers. PCR primers and protocols were successfully designed for amplification of nine microsatellite loci. Three of these loci are polymorphic and were genotyped in all animals sampled (Table S2). A total of 16 polymorphic markers was hence used in the study. Four individuals did not yield usable DNA and the total number of genotyped individuals used for the study was 188 out of the 192 collected. Of the overall possible genotypes (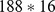), 91% were successfully scored. Among the 16 microsatellites analyzed, D136 was the most variable locus (Number of alleles, N*_a_* = 13; Observed Heterozygosity, H*_o_* = 0.690) while Z13 and CycCyc9 were the least variable (N*_a_* = 4; H*_o_* = 0.094 and N*_a_* = 3; H*_o_* = 0.197 respectively; Table S1 and Table S2).

We did not find any evidence for Linkage Disequilibrium (LD) between microsatellite markers. Of the 2760 pairwise comparisons between loci we found significant gametic disequilibrium in only 12 instances (∼0.5%) a value that does not deviate significantly from random expectation at 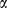 = 0.05. A single sampling site exhibits significant deviation from Hardy Weinberg Equilibrium (HWE; Site 12, Table 1). This site is actually a peninsula with three distinct patches of appropriate iguana habitat. Considering the short geographic distance between each of these patches and the fact that they are actually connected by land, collections made in these three patches were treated as a single site during analysis. One explanation for the departure from HWE seen at Site 12 would be the presence of fine scale genetic structure within the peninsula. An ad hoc analysis of individuals sampled in each of the three habitat patches (10 iguanas from the north patch, 25 iguanas from the middle patch and one iguana from the south patch) was conducted to test this hypothesis. Individuals from the north patch showed a slight but non-significant excess of homozygotes (F*_is_* = 0.087, p = 0.193). Individuals from the middle patch showed a slight but non-significant excess of heterozygotes (F*_is_* = −0.014, p = 0.626). An overall analysis of deviation from HWE using GENEPOP on the Web 4.2 (see Materials and Methods) revealed no significance (p = 0.053 north patch; p = 0.074, middle patch). This lack of significance may reflect diminished sample sizes. However, the fact that directionality of deviation from HWE differs between the two samples suggests these deviations may simply reflect sampling variance. The deviation from HWE and deficit of homozygosity detected at Site 12 may also reflect some fine scale genetic structuring (Wahlund effect). Other explanations considered include the presence of null alleles, non-random mating and heterozygote advantage. However, the presence of genotyping artifacts and null alleles was not detected during analysis with Microchecker. The small sample sizes for each patch prevent a more detailed investigation into the non-random mating and the heterozygote advantage hypotheses.

### Population structure and variability analyses

Individual assignment tests suggest three clusters based on the Evanno method [18] (Figure 1b-c and Figure S1). Individuals from Site 1 to Site 4 were grouped in a single cluster (green, Figure 1b-c; North cluster hereafter). Specimens collected from Site 5, Site 6, Site 8 and all samples from Alcorine Cay and Mangrove Cay constitute the second cluster (red, Figure 1b-c; Central cluster hereafter). The remnant individuals captured south of South Bight, including Site 17, compose the third cluster (blue, Figure 1b-c; South cluster hereafter). All clusters were characterized by the presence of private alleles (Table 2). The South cluster was characterized by the highest value of Expected Heterozygosity (H*_e_* = 0.616, s.d. = 0.177), though the Central cluster was the only group polymorphic at all 16 loci and with the highest score of Allelic Richness (A*_r_* = 3.842; Table 2).

**Table 2:** Summary statistics for the clusters identified in the individual assignment test (STRUCTURE).

Cluster	N	L	H*_o_*	H*_e_*	A*_r_*	P*_A_*
North	10	13	0.446(0.203)	0.599(0.147)	3.042	7
Center	117	16	0.540(0.223)	0.580(0.223)	3.842	16
South	61	15	0.558(0.184)	0.616(0.177)	3.663	6

Cluster name, number of individuals per cluster (N), number of polymorphic loci per cluster (L), observed heterozygosity (H*_o_*(s.d.)), expected heterozygosity (H*_e_*(s.d.)), allelic richness (A*_r_*) and number of private alleles per cluster (P*_A_*).

### AMOVA and Gene Flow

Analysis of molecular variance (AMOVA) among the genetic clusters resulted in a F*_st_* value of 0.117 (p
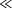
0.01). All pairwise comparisons among clusters revealed significant differences (Table 3). Results for the Bayesian estimation for non-symmetrical rates of gene flow are reported in Table 4. The proportion of individuals originating from within each identified cluster varied from 94.5 to 98.7%, with the highest value found in the Central cluster. Each independent run of BAYESASS converged towards similar values of logProb despite different starting seeds. Moreover, visualization of the MCMC trace output confirmed mixing and movement in the parameter space (Figure S2) and the posterior probability values of migration obtained from the run with the lowest estimate of Bayesian deviance [19], [20] suggests strong isolation for all the inferred clusters.

**Table 3:** Pairwise F*_st_*.

Pairwise F*_st_*	North	Center	South
**North**	—	p 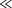 0.01 ***	p 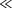 0.01 ***
**Center**	0.273	—	p 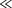 0.01 ***
**South**	0.234	0.072	—

Table shows F*_st_* values and significance level of each pairwise comparison (*** = significance at p = 0.01).

**Table 4:** Matrix of inferred gene flow between genetic clusters.

Gene Flow	North	Center	South
**North**	0.945 (−0.034)	0.025 (−0.024)	0.030 (−0.027)
**Center**	0.003 (−0.003)	0.987 (−0.008)	0.010 (−0.007)
**South**	0.005 (−0.005)	0.015 (−0.012)	0.980 (−0.013)

Values in the form m*_ij_* represent the proportion of individuals in the i*_th_* population that originated from the j*_th_* population per generation. Values in parentheses are standard deviations of the posterior probability distributions.

## Discussion

We investigated genetic structure of the endangered Andros Iguana to test the hypothesis that rates of gene flow across the island are high. The results of our study indicate that *Cyclura cychlura cychlura* does not represent a single, large panmictic population as previously inferred. That is, gene flow is much lower than estimates based on field observations would suggest.

Managing isolated local populations as a single entity, without considering metapopulation dynamics and patterns of genetic diversity, can be dangerous [21]–[25]. Although the importance of barriers to gene flow is highly dependent on the species ecology [26], fine scale genetic structure analyses demonstrated that perceived rates of gene flow may not reflect realized ones, despite the apparently high dispersal potential of a species [10], [27], [28]. Iguanas in the genus *Cyclura* tend to experience genetic structuring, presumably because these large-bodied, terrestrial lizards have difficulties dispersing across certain types of physical barriers and establishing viable populations, or because they are tightly associated with local selective pressures such as the availability of forage. Examples include *C. carinata* in the Turks and Caicos Islands [29], [30] and *C. cychlura figginsi* and *C. cychlura inornata* in the Exuma islands chain, Bahamas [31]. By contrast surveys and field observations of the Andros Rock Iguana suggested high dispersal capability [12], [14]. In particular, iguana hatchlings have been observed dispersing across a variety of different terrestrial habitats on Andros (pine woodland, shrubland and mangroves) [14] as well as water channels [12].

Our results reject the initial hypothesis of a panmictic iguana population on Andros and are indicative of historically limited patterns of gene flow. We foresee two possible explanations that could account for high dispersal yet interrupted gene flow. First, high overland dispersal rate may attenuate over narrow waterways and not extend over broader channels with stronger water currents. This scenario is in line with evidence indicating geography as an important component in lizard isolation and differentiation [32]. Second, dispersal could be relatively high but successful migrant recruitment to the breeding populations could be low due to selection acting against migrants. This could occur if there is a direct cost to dispersal or if migrants are poorly adapted to local conditions.

Fine-scale genetic structuring in reptiles and particularly iguanine lizards is not uncommon [29], [30], [33], [34]. Limited gene flow has been detected even within the only iguana adapted to a marine environment, *Amblyrhynchus cristatus* [35], [36]. Our STRUCTURE analysis suggests that samples from Andros are best partitioned into three genetically-distinct clusters. It could be argued that some portion of the inferred structure reflects recent changes in population dynamics (see for example [37]). However, our results are most likely influenced by historical landscape fragmentation and not recent anthropogenic perturbations because all samples were collected in uninhabited and remote areas of Andros. Still, some portion of the inferred genetic structure may reflect biased estimates of allele frequencies due to sampling error. Small population sizes should result in greater rates of genetic drift, enhancing allele frequency differences among subpopulations, and greater fragmentation means there should be fewer opportunities for gene flow to offset change due to genetic drift. Further, reduced population size and density are responsible for small sample sizes for many locations. However, increased variation among sampling sites within groups should make it less likely for differences among groups to appear statistically significant. Moreover, the results of the individual assignment tests demonstrate that these potential biases obstructing current perspectives on historic population genetic structure cannot explain all of the genetic divergence found among sampling sites. In particular, almost all individuals assigned to a specific genetic cluster were collected from clearly defined geographic regions divided by intervening large water channels (Figure 1b-c). The geographic boundaries of the genetic clusters identified by STRUCTURE correspond well with the two bights separating Mangrove Cay and Alcorine Cay from North and South Andros. Individuals sampled from Sites 5, 6 and 8 represent the only notable exception. Despite being located on cays within Middle Bight they were grouped with the middle cluster (Figure 1b-c). This pattern is consistent with the empirical observation of individuals floating across water channels. Water currents passing through the bights run east to west (tidal dependent), and tidal flow is further influenced by the easterly trade winds. We suspect that iguanas entering the water at the north edge of Mangrove Cay could disperse passively over water in a westerly direction. Some cays in Middle Bight (e.g. Sites 5, 6 or 8) are situated in the path of potential dispersers from Mangrove Cay as Middle Bight veers south (Figure 1b-c). Cays farther north may be more difficult to reach given the tidal currents and influence of easterly trade winds and thus remain genetically isolated from the southern populations. However, our results suggest that observations of iguanas crossing water barriers [12] rarely result in successful migration.

Overwater dispersal has been documented to play an important role for colonization, particularly in lizards [38]. Our results suggest that the terrestrial ecology of *Cyclura* iguanas makes them less adapted to swim or withstand strong water currents that flow across the bights of Andros. Still, iguanas are particular good at rafting, and are known to be highly salt tolerant [39]. It is hence conceivable that dispersal occurs more frequently across narrower or slower moving channels and is highly influenced by the directionality of the water current.

Consistent with evidence suggesting historically low inferred rates of gene flow, private alleles were restricted to each of the three geographic clusters further supporting that Middle and South Bights serve as major barriers to successful iguana dispersal. Differences in genetic makeup that generate genetic structure build up over time in the form of variance in allele frequencies across subpopulations and the emergence of private alleles due to random genetic drift or mutational events [40]. Our inferred estimates of gene flow also contradict genetic homogeneity across Andros. The significant AMOVA value (F*_st_* = 0.117; p
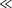
0.01) for genetic clusters identified by STRUCTURE indicates that more than 11% of the total genetic variance on Andros reflects significant differences in allele frequencies among the three clusters. This value is in accordance with other estimates of genetic isolation documented in other iguana taxa (*Amblyrhynchus cristatus* F*_st_* = 0.002–0.011, [36]; *Cyclura carinata* F*_st_* = 0.18–0.43, [29]). Pairwise estimates of genetic differentiation (Table 3) indicate that the Center and South clusters are more closely related to each other than either is to the North cluster (Central-South F*_st_* = 0.072, p
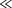
0.01; Center-North F*_st_* = 0.273, p
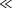
0.01) suggesting a genetic uniqueness of the North cluster. Moreover, the Bayesian inference of recent migration rates substantiates the existence of at least three distinct iguana populations on Andros (Table 4). The proportion of individuals originated locally within each identified cluster varied from 94.5 to 98.7% indicating that there is little gene flow between any of these populations. The small percentage of migrant genotypes found within each cluster could represent ancient polymorphisms retained among the genetic and geographic isolates of today.

## Conclusions

We document that *C. c. cychlura*, despite its dispersal potential, shows significant genetic structuring and that natural landscape features (i.e. water channels) influence successful migration and thus genetic structure across the island. That is not to say that anthropogenic activities are not important in further compromising the extant metapopulation dynamics on the island, and the extremely low density of individuals on North Andros (see [12] for details) is a clear example of what could happen when human development goes unregulated. Our data also suggest that, in accordance with ecological observations, the identified cluster on Alcorine and Mangrove Cays harbor significant genetic diversity relative to other populations. Genetic diversity is the fundamental requirement for adaptive evolution in response to environmental changes and should be correlated with the resilience of populations to novel environmental selective pressures [41]. In addition, reduction in genetic diversity and heterozygosity is tightly linked to inbreeding depression and can enhance the probability of extinction in stressful environments [41], [42]. Although our genetic data are not directly indicative of any enhanced adaptive potential for iguanas in the Central cluster, it would be wise to focus the limited conservation resources on the subpopulation showing higher degree of variability at neutral-nuclear markers and higher density of individuals. In 2009, the West Side National Park was expanded, in part based on ecological and population studies of *C. c. cychlura* [15]. The expanded boundaries include Alcorine Cay and segments of Mangrove Cay. Our molecular data confirm the strategic importance of the new boundaries. The extension of the national park now includes a much larger portion of the island, enhancing the future prospects for the iguanas in the central region of Andros Island. However, the prospects for the two iguana populations associated with North and South Andros are far less certain. Our data suggest that these populations, in particular the one north of North Bight, are genetically unique. Given their uniqueness and that they reside largely outside park boundaries additional efforts should be taken to ensure that these populations also receive protection.

## Materials and Methods

### Ethical statement

We thank the Bahamas Environment, Science and Technology commission for permission and permits to conduct the study. We thank the Bahamas National Trust for permission to work in the West Side National Park. The Bahamas Ministry of Agriculture issued the CITES export permits. This work was made possible through the help of Shedd Aquarium volunteer research assistants. Methodologies for this study were approved by the Shedd Aquarium research review committee.

### Study system and sample collection

Andros is the largest island in the Bahamian archipelago encompassing an area of 5,959 km^2^ and supporting a human population between 8000 and 9000 concentrated along the eastern coast [13], [43]. This subtropical island is a composite of four main islands (North Andros, Mangrove Cay, Alcorine Cay and South Andros), along with hundreds of associated cays, separated by wide (≥5 km) saline tidal channels and smaller saline creeks. The substrate consists of oolitic and bioclastic limestone, and from east to west, a thin coastal ridge (to 30 m elevation) gives way to a flat and pine-forested interior. Approximately halfway across the island in a westerly direction, forest grades into extensive shrubland, mudflats, and mangroves as the water table reaches the surface. Knapp et al. [14] provide detailed descriptions of primary plant communities from study areas.

Less than 5,000 iguanas remain on the island and when present, occur in low densities (0.5–2.5 adults/ha) [16]. Our sampling effort over the years covered the whole island. On North Andros, iguanas are rarely encountered due to habitat degradation from historic logging practices, poaching, and predation from non-native mammals [12]. Therefore, fieldwork was focused between the southern extent of North Andros Island (24° 22.103′N) to the southernmost cays associated with the island (23° 38.569′N). Accessing study sites and locating iguanas presented serious logistical challenges, as much of Andros Island is extremely remote and iguanas occur in low densities.

Tissue samples were collected from animals captured during fieldwork from 1999 to 2013 using fish-landing nets or nooses. A total blood volume of 1–2 ml was drawn from each individual via the ventral coccygeal vein using a heparinized syringe. Blood samples were stored in SDS lysis buffer (0.1M Tris-HCl pH 8.0, 0.1M EDTA, 0.01M NaCl, 2% SDS) at ambient temperature prior to long term storage at −80°C [44].

### DNA Extraction and Genotyping

Approximately 20*µl* of blood lysate was digested for five hours in a 65°C water bath with Proteinase-K (20 mg/ml) in digestion buffer (17mM Tris-HCl, 1.7mM CaCl_2_ and 50% glycerol) [45]. Following digestion, genomic DNA was extracted using an ABI PRISM—6100 Nucleic Acid Prep Station and proprietary chemistry (Applied Biosystems, Foster City, California, USA). Successful DNA extraction was assessed through electrophoretic migration in a 1% agarose gel.

A suite of 23 microsatellites developed for congeneric species were screened for positive amplification and variability [46]–[50]. Three-primer PCR [51] was performed in 10* µ*L volumes with ∼10 ng DNA, 2 mM MgCl_2_, 30 mM tricine (pH 8.4)-KOH, 50 mM KCl, 100 *µM* of each dNTP, 200 nM of reverse primer and M-13 forward primer (CACGACGTTGTAAAACGAC) labeled with fluorescent dye (HEX, NED, FAM, VIC or PET), between 40 and 150 nM forward primer with the M-13 extension, and 0.4 U of Taq DNA polymerase. Touchdown-PCR [52] profiles were set with an initial denaturation period of 5 min at 94°C followed by 10 touchdown cycles with 30 s at 94°C, 30 s at annealing temperature, and 30 s at 72°C. In touchdown protocols the initial annealing temperature is 10°C above the final annealing temperature. In each successive PCR cycle the annealing temperature drops by 1°C. The remaining 25 cycles had thermal cycling profiles of 30 s at 94°C, 30 s at 52°C, and 30 s at 72°C. A final elongation phase of 7 min at 72°C completed the PCR cycle profiles. Fragment analysis was performed on ABI 3730 capillary sequencers (Applied Biosystems) at the Arizona State University DNA Laboratory using LIZ-500 as a size standard (GeneScan — 500 LIZ Size Standard— Applied Biosystems). Genotypes were visually scored using Peak Scanner version 1.0 (Applied Biosystems).

Additional microsatellite markers were developed specifically for *C. c. cychlura*. Tandem repeat regions were identified using the subtractive hybridization method of Glenn and Schable [53]. Digested DNA was enriched for eight oligonucleotide repeats (AC)_15_, (AG)_15_, (AAC)_10_ and (AGG)_10_. Enriched PCR products were cloned using the pGEM-T cloning kit (Promega Madison, WI) with color screening. Ninety-three color positive (i.e. white) colonies were suspended in 50* µ*L T.E. buffer (10 mM Tris-HCl pH 8.0; 0.1 mM EDTA, pH 8.0). A PCR was used to screen for inserts of suitable size for sequencing. Reactions were 10* µ*L in volume and contained 0.5* µ*L template DNA, 0.3* µM* each of primers pUC-M13F and pUC-M13R (Integrated DNA Technologies), 1X GoTaq Flexi Buffer (Promega Corporation, Madison, WI), 2mM MgCl2 160* µM* of dNTPs and 0.5 units of GoTaq Flexi DNA polymerase (Promega Corporation, Madison, WI). PCR profile consisted of 3-min at 95°C, followed by 35 cycles of 95°C for 30 s, 50°C for 30 s, 72°C for 1.5 min and lastly a single extension period at 72°C for 7 min. Amplicons were electrophoresed in 1.5% agarose TBE gels and visualized by ethidium bromide and UV light. Clones that exhibited a single amplified band of 500–1000 base pairs were cleaned with 16 U of Exonuclease I and 3 U of Antarctic Phosphatase (New England Biolabs, Ipswich, MA) followed by ethanol precipitation. Cleaned PCR products were sequenced using the pUC-M13F primer, Big Dye Terminator v. 3.1 Cycle Sequencing Kit (Applied Biosystems, Foster City, CA) in 10* µ*L reactions including 0.5* µ*L Big Dye, 0.3* µ* primer, 0.87X sequencing buffer (Applied Biosystems, Foster City, CA), and 2* µ*L of cleaned PCR product. Sequence reactions were cleaned using columns packed with Sephadex and then electrophoresed at the Arizona State University DNA Lab. Microsatellites were identified using Tandem Repeats Finder [54] and primers designed using Primer3 [55].

### Data analysis

We first test for evidence of linkage disequilibrium between pairs of loci using an exact probability test in GENEPOP on the Web 4.2 [56], [57]. We set the Markov-Chain parameters to compute 1000 dememorisation steps, 100 batches and 1000 iterations per batch. Observed and expected heterozygosities (H*_o_* and H*_e_*) were calculated according to Nei [58] and using ARLEQUIN 3.5 [59]. We additionally tested for any significant departure from Hardy Weinberg Equilibrium (HWE), following Guo and Thompson [60] using GENEPOP on the Web 4.2 [56], [57]. We used 5000 dememorization steps, 100 batches and 5000 iterations per batch in the Markov Chain. The sequential Bonferroni correction was used to adjust significance thresholds [61] when necessary.

### Population structure

To test our hypothesis that iguana populations across Andros are genetically homogeneous due to high rates of dispersal and gene flow, we performed a Bayesian-based individual assignment test using STRUCTURE v.2.3.4 [62]. The program assigns individuals to inferred populations based on the posterior probability that a certain genotype is sampled from a modeled allelic distribution. An ancestral iguana population was assumed to have recently split due to rising sea levels associated with the current interglacial period [46]. We assumed an admixture ancestry model with correlated allele frequencies and no prior information on sampling locations. A total of 10^6^ MCMC iterations were calculated, and the first 100,000 replicates were discarded as burn-in. We assumed K putative populations ranking in number from one to 10 and performed 10 iterations of the MCMC sampling procedure for each K value. The most likely number of clusters was estimated using the Evanno method, based on the second order of difference in likelihood function of K (i.e. 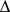
K) and implemented in the web tool STRUCTURE - HARVESTER [18],[63].

Overall genetic variability across putative genetically isolated regions was also quantified. For each population, identified by Bayesian clustering, the percentage of polymorphic loci and private alleles (P*_a_*) were calculated using the GENALEX 6.5 plug-in for Excel [64]. ARLEQUIN 3.5 [59] was used to calculate observed and expected heterozygosities (H*_o_* and H*_e_*). Due to the differences in sample sizes across different clusters, and given that allelic richness should be a function of sample size, we used an allelic richness index (A*_r_*) that rarefies the number of alleles according to the number of genes examined in the smallest population [65]. A*_r_* was calculated using HIERFSTAT, a module designed for use in the R statistical software [65], [66].

### AMOVA and gene flow

To test the significance of any genetic isolates and to infer the rate of gene flow among hypothesized clusters, hierarchical AMOVAs were performed using ARLEQUIN 3.5 [59], [67], [68]. The proportion of genetic variation attributable to STRUCTURE grouping (F*_st_*) was quantified and tested for statistical significance. We also performed a pairwise comparison (pairwise-F*_st_*) in order to estimate migration rates between pairs of clusters.

Inferences of gene flow based on F statistics (4N*_e_*m = 1/F*_st_*-1; [69], [70]) are relatively insensitive to rare alleles and in general are based on rather simplistic assumptions [71], [72]. We hence used the Bayesian approach developed by Wilson and Rannala in BAYESSASS 3.0 [72] to infer migration rates. The program uses genotypic data and MCMCs to more accurately infer recent patterns of gene flow. This approach does not explicitly calculate the number of migrants, unless the analyzed populations have equal numbers of individuals, but it returns the proportion of immigrants within each population, allowing for indirect estimates of gene flow [72]. We performed five independent analyses. In each run the program computed 10^7^ MCMCs and discarded 10^6^ chains as burn-in. Chains were sampled every 2,000 generations. To ensure sufficient mixing of the MCMCs and to improve the coverage of the probability space we adjusted the acceptance rate for estimated allele frequencies and inbreeding coefficients. We hence increased the mixing parameters for both allele frequencies (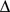
A) and inbreeding coefficient (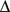
F) to 0.30 as suggested by the authors [72]. Each independent run started with a different random seed. Mixing and convergence of MCMCs were visually assessed using TRACER 1.6 [73]. Among the five independent runs, we chose the one with lowest Bayesian deviance in the logProb calculated using the r-function provided by [19] and as suggested in [20].

## Supporting Information

Figure S1
**The most likely number of populations as identified by the Evanno method** [18] **.** K = 3 showed the highest DeltaK value for all values of K ranging from 1 to 10 calculated using DeltaK = m(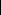
L"(K)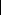)/sd(L(K)).(TIFF)

Figure S2
**BAYESASS 3.0 Trace-plot and analysis parameters for the run with the lowest Bayesian deviance.** The X-axis is log probability. The Y-axis is number of Bayesian iterations. The gray shaded trace represents the burn-in. Random seed  = 445; MCMC iterations  = 10,000,000; burn-in  = 1,000,000; Sampling interval  = 2,000; Mixing parameters: (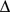
M = 0.1, 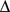
A = 0.3, 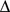
F = 0.3); Bayesian Deviance  = 12,984.72.(TIFF)

Table S1 **Molecular marker information for loci characterized in congeners of** ***C. c. cychlura*** **.** Name, reference, size range number of alleles (N*_a_*) observed heterozygosity (H*_o_*(s.e.)) and expected heterozygosity (H*_e_*(s.e.)) in *C. c. cychlura*. Summary statistics are based on the total sample.
(PDF)

Table S2 **Molecular marker information for newly characterized loci.** Name, GenBank Accession number, primer sequences, repeat motifs, annealing temperatures, size ranges, number of alleles (N*_a_*), observed heterozygosity (H*_o_*(s.e.)) and expected heterozygosity (H*_e_*(s.e.)). Summary statistics are based on the total sample.
(PDF)

Table S3 **Genotyped individuals.** Excel spreadsheet containing the complete list of genotyped individuals used in this study.
(XLSX)
