# Supplementary figures and images for: Inferred vs Realized Patterns of Gene Flow: An Analysis of Population Structure in the Andros Island Rock Iguana
Source: PLoS One. 2014 Sep 17;9(9):e106963. doi: 10.1371/journal.pone.0106963 (PMC4167547; doi:10.1371/journal.pone.0106963)

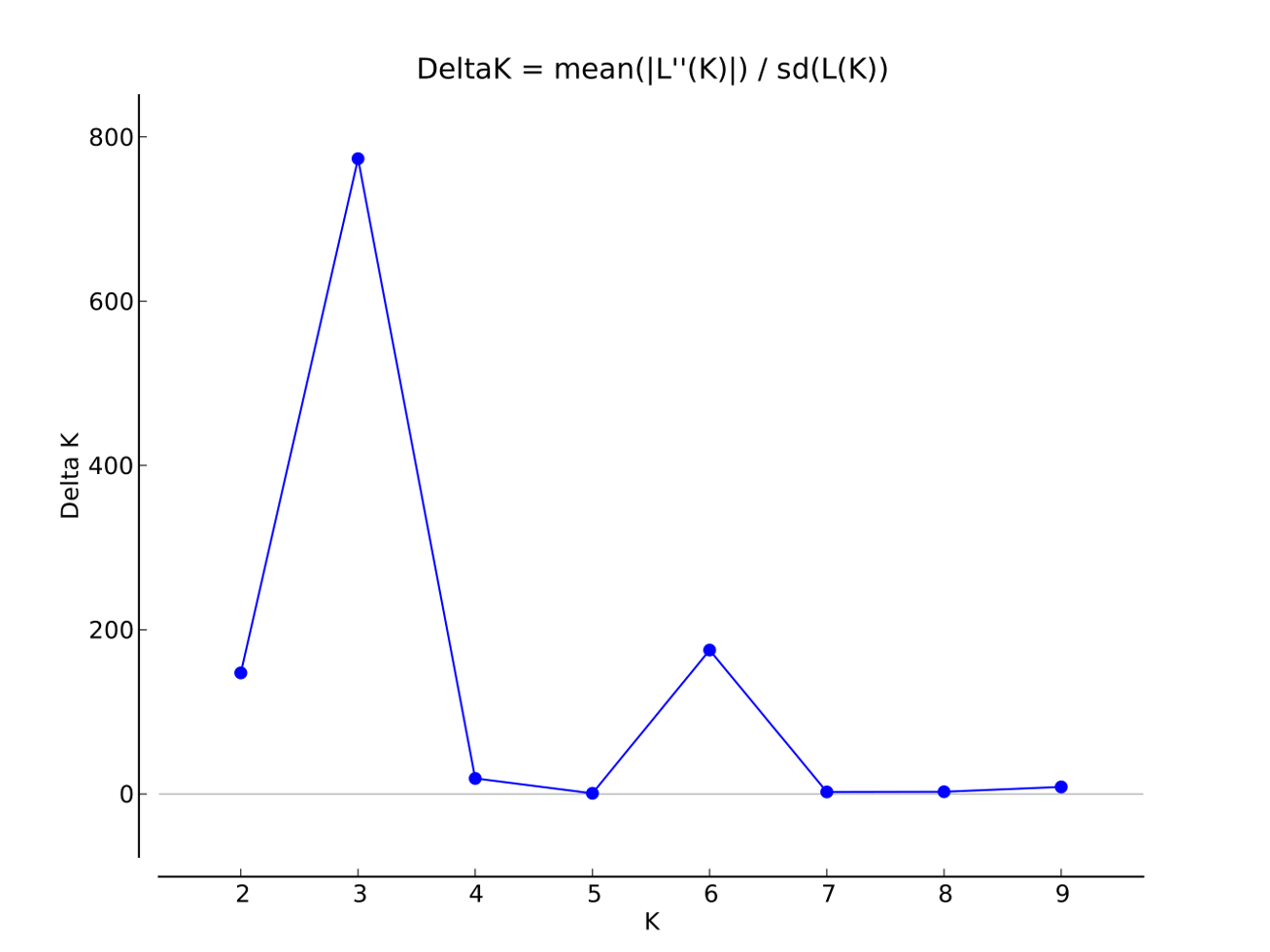

Supplement: Figure S1 — The most likely number of populations as identified by the Evanno method [18] . K = 3 showed the highest DeltaK value for all values of K ranging from 1 to 10 calculated using DeltaK = m(L"(K))/sd(L(K)). (TIFF) [file pone.0106963.s001.tiff]

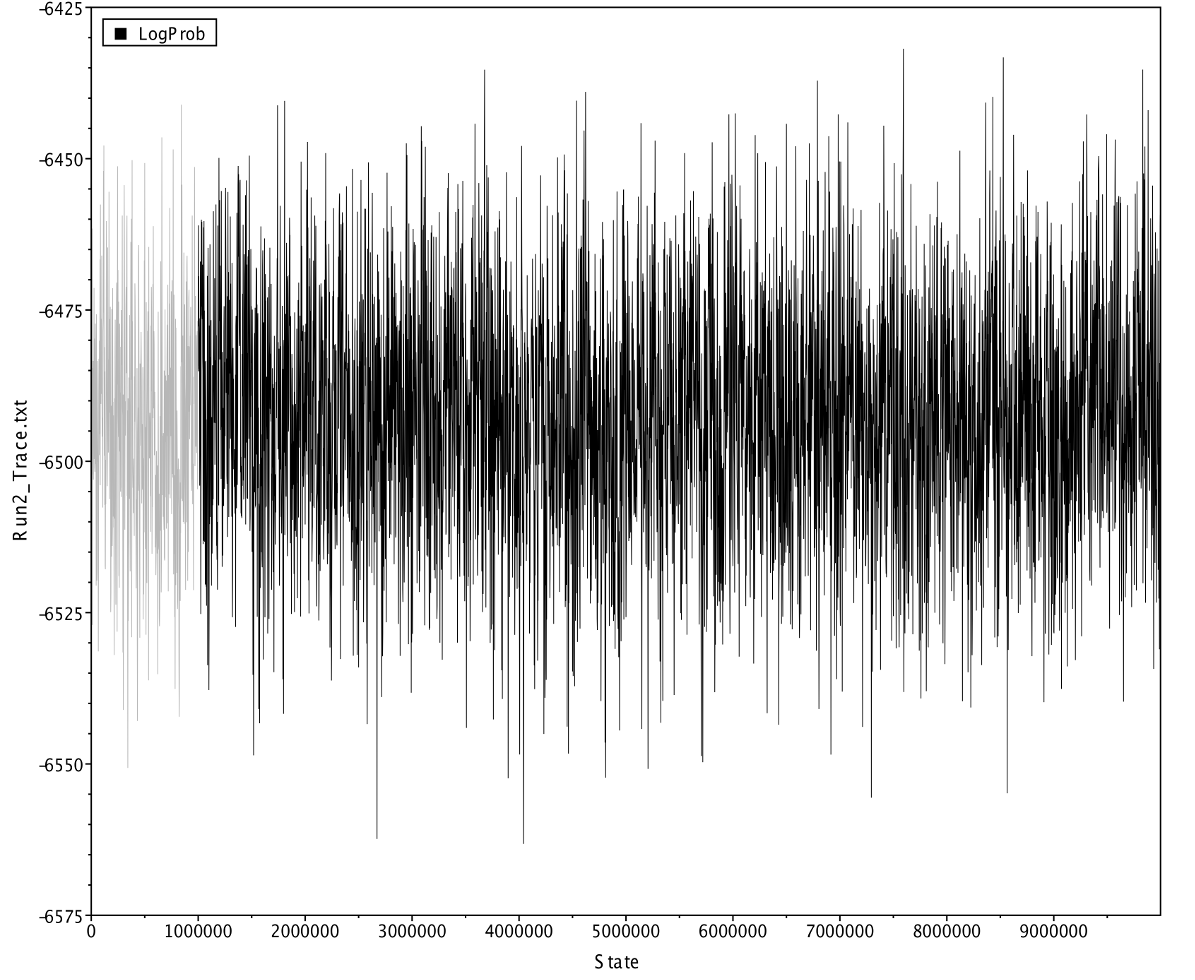

Supplement: Figure S2 — BAYESASS 3.0 Trace-plot and analysis parameters for the run with the lowest Bayesian deviance. The X-axis is log probability. The Y-axis is number of Bayesian iterations. The gray shaded trace represents the burn-in. Random seed = 445; MCMC iterations = 10,000,000; burn-in = 1,000,000; Sampling interval = 2,000; Mixing parameters: (M = 0.1, A = 0.3, F = 0.3); Bayesian Deviance = 12,984.72. (TIFF) [file pone.0106963.s002.tiff]
