# Supplementary material for: Inferred vs Realized Patterns of Gene Flow: An Analysis of Population Structure in the Andros Island Rock Iguana
Source: PLoS One. 2014 Sep 17;9(9):e106963. doi: 10.1371/journal.pone.0106963 (PMC4167547; doi:10.1371/journal.pone.0106963)
Supplement: Table S1 — Molecular marker information for loci characterized in congeners of C. c. cychlura . Name, reference, size range number of alleles (Na) observed heterozygosity (Ho(s.e.)) and expected heterozygosity (He(s.e.)) in C. c. cychlura. Summary statistics are based on the total sample. (PDF) [file pone.0106963.s003.pdf]

Table S1

**Name**	**Reference**	**Size Range**	**N_a**	**H_o**	**H_e**
F478	[45]	208-226	9	0.651 (0.065)	0.490 (0.031)
F519	[45]	331-355	9	0.577 (0.069)	0.494 (0.044)
C6	[47]	125-145	8	0.646 (0.059)	0.512 (0.039)
C124	[47]	210-248	9	0.677 (0.062)	0.571 (0.043)
D136	[47]	162-210	13	0.690 (0.067)	0.552 (0.048)
Z13	[46]	269-283	4	0.094 (0.036)	0.177 (0.039)
Z65	[46]	160-181	5	0.544 (0.067)	0.477 (0.051)
Z99	[46]	122-135	5	0.219 (0.055)	0.226 (0.042)
Z106	[46]	179-192	6	0.530 (0.067)	0.499 (0.044)
Z154	[46]	214-231	4	0.267 (0.068)	0.333 (0.054)
Z494	[46]	197-214	5	0.466 (0.066)	0.414 (0.053)
CCSTE02	[48]	288-299	6	0.671 (0.064)	0.515 (0.046)
CIDK177	[49]	258-285	12	0.587 (0.074)	0.502 (0.058)

Table S1. Molecular marker information for loci characterized in congeners of *C. c. cychlura.* Name, reference, size range number of alleles (N_a) observed heterozygosity (H_o(s.e.)) and expected heterozygosity (H_e(s.e.)) in *C. c. cychlura*. Summary statistics are based on the total sample.
