# Supplementary material for: Inferred vs Realized Patterns of Gene Flow: An Analysis of Population Structure in the Andros Island Rock Iguana
Source: PLoS One. 2014 Sep 17;9(9):e106963. doi: 10.1371/journal.pone.0106963 (PMC4167547; doi:10.1371/journal.pone.0106963)
Supplement: Table S2 — Molecular marker information for newly characterized loci. Name, GenBank Accession number, primer sequences, repeat motifs, annealing temperatures, size ranges, number of alleles (Na), observed heterozygosity (Ho(s.e.)) and expected heterozygosity (He(s.e.)). Summary statistics are based on the total sample. (PDF) [file pone.0106963.s004.pdf]

Table S2

**Name/Acc.**	**Sequence(5' to 3')**	**Motif**	**T°**	**Range$_a$**	**N$_a$**	**H$_o$**	**H$_e$**
CycCyc9	F: TGCAGTTTGTCCTTTGTTGC	(GT)	52	206-221	3	0.197 (0.05)	0.164 (0.04)
KF646798	R: CTTGAGCCACCCATTTCTTG						
CycCyc16	F: TGGCAACCCTGTAAATCCTC	(CA)	52	167-187	5	0.553 (0.078)	0.444 (0.042)
KF646799	R: TGAGACTGGAAGATTGCCTTG						
CycCyc31	F: TGGCCCAAGCATTAATAAAA	(CTA)	52	170-197	5	0.311 (0.082)	0.253 (0.053)
KF646800	R: CATGGGAGGGAGGGAATAAT						

Table S2. Molecular marker information for newly characterized loci. Name, GenBank Accession number, primer sequences, repeat motifs, annealing temperatures, size ranges, number of alleles (N_a), observed heterozygosity (H_o(s.e.)) and expected heterozygosity (H_e(s.e.)). Summary statistics are based on the total sample.
